# The diagnostic contribution of CT volumetric rendering techniques in routine practice

**DOI:** 10.4103/0971-3026.63043

**Published:** 2010-05

**Authors:** Simone Perandini, N Faccioli, A Zaccarella, TJ Re, R Pozzi Mucelli

**Affiliations:** Department of Radiology, G.B. Rossi Hospital, University of Verona, Verona, Italy

**Keywords:** Computed tomography, CT, volume rendering, VR, MIP, MinIP, shaded surface display, virtual endoscopy, curved plane reconstructions

## Abstract

Computed tomography (CT) volumetric rendering techniques such as maximum intensity projection (MIP), minimum intensity projection (MinIP), shaded surface display (SSD), volume rendering (VR), and virtual endoscopy (VE) provide added diagnostic capabilities. The diagnostic value of such reconstruction techniques is well documented in literature. These techniques permit the exploration of fine anatomical detail that would be difficult to evaluate using axial reconstructions alone. Although these techniques are now widely available, many radiologists are either unfamiliar with them or do not fully utilize their potential in daily clinical practice. This paper is intended to provide an overview of the most common CT volumetric rendering techniques and their practical use in everyday diagnostics.

## Introduction

The widespread introduction of multidetector computed tomography (MDCT) has revolutionized the field of computed tomography (CT). This revolution can be attributed to three primary properties of MDCT: its ability to produce a vast quantity of volumetric data in a reduced amount of time, the high resolution, and the ability to create isotropic voxel data and, consequently, reliable multiplanar and three-dimensional (3D) reconstructions.

Diagnostic approaches that rely solely on axial reconstructions of MDCT data, as was the norm with older CT scanners, often are insufficient for formulating an accurate diagnosis or for documentation of clinical cases. Specialized 3D reconstruction techniques permit the visualization of anatomical details, which would be difficult to evaluate using axial reconstructions alone. Such details may require the use of oblique or curved reconstructions (e.g., to visualize the path of a winding vessel or duct), or more complex methods, such as maximum intensity projection (MIP), minimum intensity projection (MinIP), surface-shaded volume rending (SS-VRT), and virtual endoscopy. Furthermore, some lesions such as some pulmonary nodules can only be rapidly and reliably identified through the use of such specialized techniques. These techniques also greatly increase the sensitivity for localizing smaller lesions and thus improving the overall accuracy for localizing lesions, which is essential for tumor staging and treatment follow-up.[1,2]

**Figure 1 (A,B):**
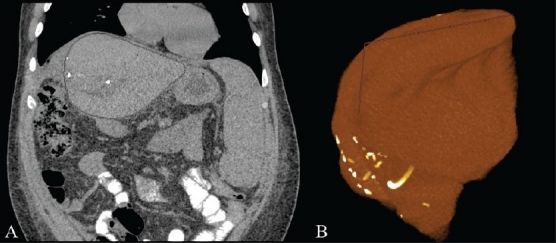
Coronal CT scan (A) and segmented volume rendered (B) images of a partially resected liver. With this technique, it is possible to determine the volume of the residual liver in milliliters, essential clinical information for the post-operative patient

Three-dimensional reconstructions are obtained by means of dedicated computer software that can handle the volumetric data of CT. Although 3D reconstruction software varies from one CT scanner manufacturer to another, they all tend to merge the routine diagnostic console and 3D reconstruction workstation. The integration of 3D reconstruction utilities into the standard bidimensional diagnostic software has vastly increased the number of operations possible on each exam data, greatly increasing the perceived complexity of CT diagnosis. There is a well-founded feeling that the use of 3D reconstructions greatly increases total exam evaluation time. However, reports in literature show how using 3D reconstruction techniques for examining volumetric data is effective and also improves the speed of interpretation, recognition, and description of specific clinical conditions.[3–5] Such reconstruction techniques are of particular importance for the analysis of subspecialty exams. For example, one study of preoperative liver transplant patients has shown how volumetric reconstruction techniques are a crucial element in therapeutic decision-making, allowing the discrimination of potential transplant candidates and the calculation of important parameters such as healthy liver volume [Figure 1] and portal hypertension;[6] such data are not available with classic imaging techniques.

This article is intended as an overview of CT volumetric reconstruction techniques and their application in some diverse practical clinical situations.

## Algorithms and Reconstruction Techniques

**Figure 2:**
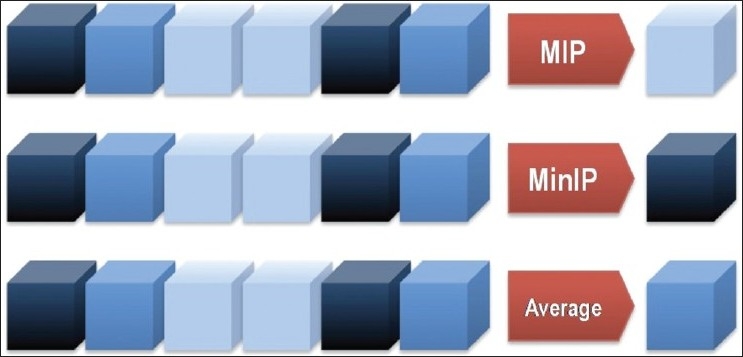
Model to demonstrate the functionality of three fundamental CT algorithms. In this model, a set of voxels to process is represented as a line of blue-shaded cubes. The lighter the blue shade, the higher the Hounsfield value represented. The MIP algorithm visualizes the highest Hounsfield value (lightest) for a set of voxels, Minimum Intensity Projection (MinIP) shows the lowest Hounsfield value (darkest), and Average Intensity Projection (AIP) shows the median Hounsfield value

An algorithm is an effective method for problem solving using a finite sequence of instructions. In computer science, algorithms process numbers using mathematic operations. In radiology too, algorithms process numbers representing tissue information (radiological density in the case of conventional x-rays and CT, acoustic response in the case of ultrasound, and signal intensity in the case of MRI) and primarily generate images by mapping quantitative results as a gray-scale or, occasionally, color parameter. The creation of new and customized visual representations by applying mathematical algorithms to the original data is called a reconstruction technique. Many techniques have been developed to post-process CT volumetric data. The simplest techniques extract one single parameter of the volumetric data and produce two-dimensional (2D) reconstructions highlighting a desired structure. The most commonly used of these simple techniques are: the average projection, MIP, and MinIP [Figure 2]. More advanced operations process volumetric data, creating an elaborate 3D model, which can be further manipulated for visualizing complex structures. Such operations are used in SS-VRT and virtual endoscopy techniques. Other types of techniques generate data from a semi-automated input given by the operator and are fundamental for curved plane reconstructions.[3]

## Specific Algorithms

### MIP

**Figure 3 (A,B):**
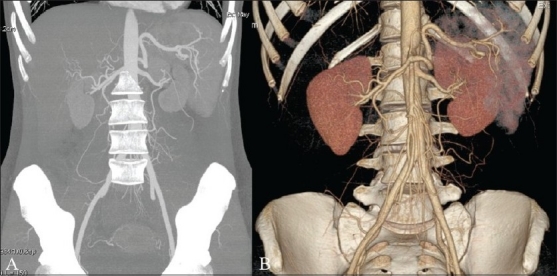
Maximum intensity projection (A) and volume rendered (B) images of the abdomen show the renal arteries of a healthy potential kidney donor

MIP is a data visualization method that enables detection of highly intense structures. The algorithm uses all the data in a volume of interest to generate a single bidimensional image.[4] Such an algorithm is rather simple: for each XY coordinate only the pixel with the highest Hounsfield number along the Z-axis is represented so that in a single bidimensional image all dense structures in a given volume are observed. For example, it is possible to find all the hyperdense structures in a volume, independently of their position [Figures 2 and 3].

The MIP algorithm is diagnostically useful because it can readily distinguish structures that are hyperdense with respect to surrounding tissues. As an example, when this reconstruction algorithm is used with data representing the thorax during the arterial contrast phase, a single image with all the arterial vessels present in the volume studied is generated. Detecting voxels with higher density enables the radiologist to better understand the extension and morphology of some structures, such as vessels, nodules, calcifications, surgical clips, foreign bodies, etc., and significantly reduces the time needed to analyze complex structures in different planes and with a non-linear course. This method is particularly useful in daily practice to detect small lung nodules,[5,6] which can easily be distinguished from other dense structures in the lungs, with the air present in the alveoli acting as a natural contrast agent.

Since it is possible to generate in real-time MIP images with a desired slice thickness, it is common to use this technique to render partially superimposed MIPs while scrolling through the whole scan volume in both directions. In this way, the hyperdense nodules are represented for longer periods of time because they are present in more than one reconstructed MIP image and always displayed at maximum size. In this regard, MIP images have proven to be a faster and more sensible method than standard viewing of axial images for detecting small lung nodules.[1,2]

MIP is also used in angiography because it can detect the opacity of a vessel against tissues with lower densities and, therefore, can follow the complete course of the structures containing contrast agents even if they are tortuous, as is the case with gastric varicose veins.[7,8] In particular, it is possible to acquire various MIP images, each one obtained from a slightly more angulated point of view with respect to the preceding one so that the perception of rotation is created; this is especially useful for the analysis of complex vascular structures. Such a method enables more accurate detection of the position in space of eventual enlargements and defects.

### MinIP

**Figure 4 (A,B):**
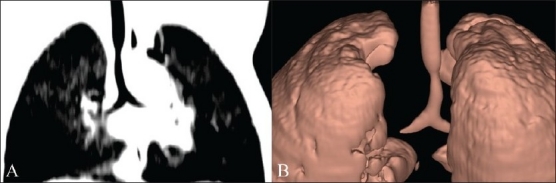
Osteocondroplastic tracheopathy in a pediatric subject. MIP image (A) and a posterior projection volume rendered image (B) show the length of the tracheal stenosis (arrow)

Minimum intensity projection (MinIP) is a data visualization method that enables detection of low-density structures in a given volume. The algorithm uses all the data in a volume of interest to generate a single bidimensional image.[3] The MinIP algorithm is almost identical to the MIP algorithm but, in the case of MinIP, for each XY coordinate only the lowest Hounsfield value along the Z axis is represented. In this way, only the most hypodense structures of the volume are represented [Figure 2], regardless of their plane of location. For example, by performing a MinIP mapping of the thorax before administration of contrast, an image of the bronchial tree can be generated since the bronchi, being air-filled, are the least dense structures of the thorax [Figure 4]. The possibility of highlighting hypodense voxels helps the radiologist to better understand the extent and morphology of some types of structures (airways, vessels, ducts, trapped air, etc.), often significantly reducing the time required to analyze complex multiplanar or nonlinear structures.[7]

The MinIP algorithm is particularly useful for analyzing the bile tree and pancreatic duct, which are hypodense compared to surrounding tissue, especially in the pancreatic and portal phase of contrast agent administration.[8]

### SS-VRT and virtual endoscopy

Shaded surface display volume rendering (SS-VRT) is a technique that creates a 3D visual illustration of CT volumetric data for display from any desired perspective.[9] SS-VRT images provide a sensation of three-dimensionality that is significantly superior to other volume rendering techniques.[3] The SS-VRT techniques are quite complex and processor intensive but are now becoming commonplace. These techniques typically select voxels to be included in a surface rendering based on a selected range of Hounsfield values. By properly choosing the Hounsfield range, different types of tissues can be selected: parenchyma, bone, airways, and vessels. By analyzing a combination of Hounsfield ranges, a volume of CT data can be segmented into several of these tissue types. These techniques then calculate the location of surfaces separating tissue types. The surface information is then used to calculate a perspective visualization based on selectable observer position and light source positioning (ray-tracing techniques).

**Figure 5 (A,B):**
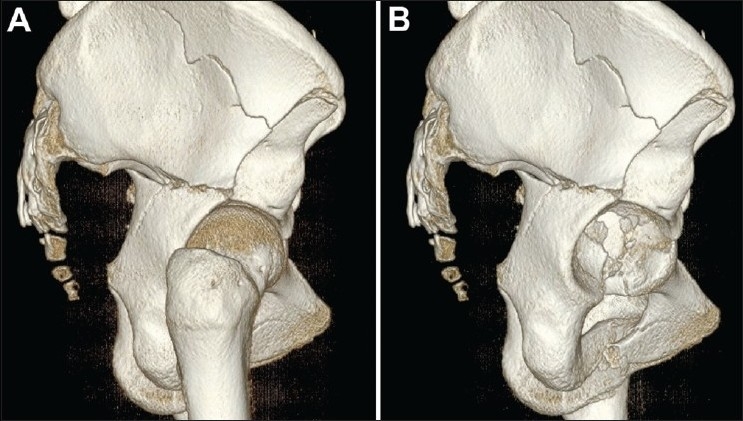
Disarticulation. Shaded surface volume rendering of bone tissue in a complex pelvis fracture without (A) and after disarticulation (B) of the femur show that the complex fracture involving the acetabulum is better appreciated after disarticulation

SS-VRT typically exploits real-time user manipulation of view perspective and virtual light sources to zoom in and highlight minute anatomical details. These techniques are particularly apt for studying fine details such as articular bone surfaces. The principle diagnostic utility of SS-VRT techniques is its ability to represent with great detail structures of a specific density. With successive interactive steps of exclusion/inclusion of different tissue types and resizing/trimming of the region of interest, surfaces that would otherwise be very difficult to visualize can be visualized. This progressive process of selection of tissue type/regions of interest is particularly apt for revealing and studying articular surface fracture lines, which often remain hidden behind adjacent bone surfaces. These surface modeling techniques typically provide a disarticulation utility that, essentially, renders invisible (by simply not generating its surfaces) one or more of the bone structures of a joint. By disarticulation, one can virtually “decompose” a bone joint and study the internal surfaces of the remaining structures for fractures [Figure 5].

**Figure 6:**
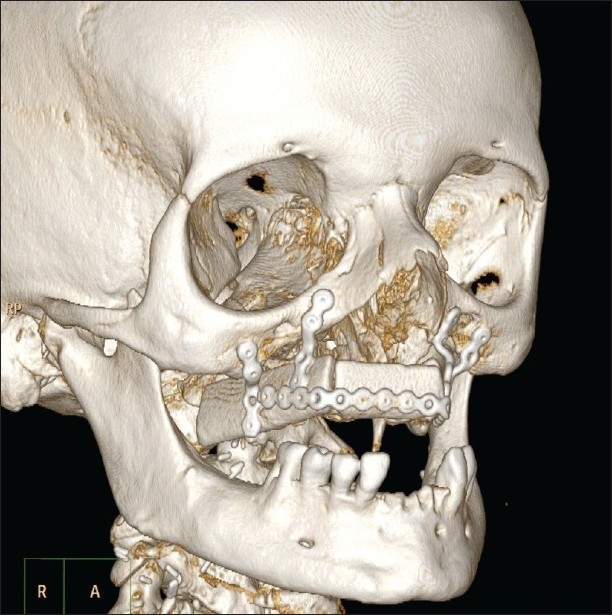
Shaded surface volume rendering of bone tissue in a maxillofacial patient undergoing reconstructive surgery of the maxilla shows the position of the metallic plaques and prostheses, whose position and relationships would be difficult to interpret using only axial scans

However, the potential of these surface modeling techniques is by no means limited to diagnosing small hidden fracture lines. They also are of great use in documenting more extensive lesions, such as multiple rib fractures, or complex fractures with multiple dislocated fragments as may be seen in maxillofacial trauma or even distal limb fractures[10–15] [Figure 6].

A particularly important application of surface rendering techniques in CT is virtual endoscopy. The surface rendering technique can be used to simulate an endoscopic exam by locating a point of view inside a hollow organ lumen. When compared to traditional endoscopy, virtual endoscopy has the advantage of being noninvasive and capable of virtually exploring regions inaccessible to an endoscopic device, such as areas distal to a lumen obstruction. Currently, virtual endoscopy has been successfully used for studying a great number of anatomical locations, the most common being the colon [[Fig F0007]], respiratory tract, auditory canal, and urinary tract.[11,12] Virtual colonoscopy is used in selected cases where the patient is unable to undergo a traditional endoscopy, for example, in patients with a high risk of perforation, or when the path of the endoscopic probe is limited by a stenosis or luminal obstruction, or when sedation is contraindicated. It has also been demonstrated that virtual gastroscopy provides a more accurate Bormann gastric cancer staging and greater sensitivity in detecting early gastric cancer than cross-sectional CT studies of the stomach.[13] It has also successfully been used to explore the nasal cavity when planning surgery for maxillofacial pathologies,[14,15] as well as for performing virtual laryngoscopies.[16]

**Figure 7 F0007:**
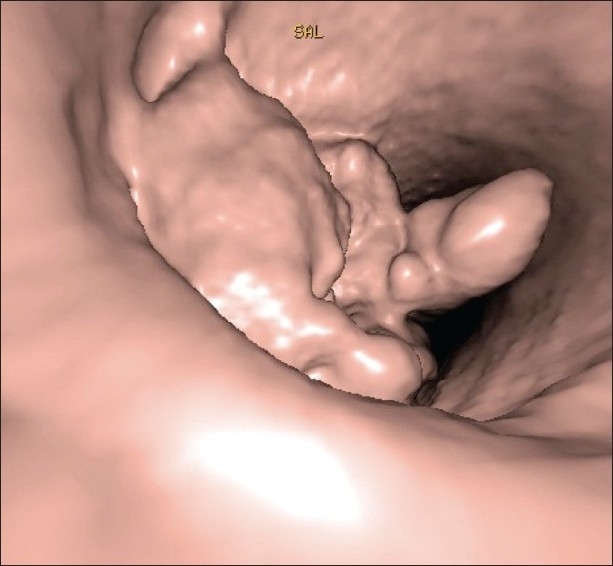
Virtual colonoscopy. Polyps (arrows) are seen protruding into the lumen of the colon, in this shaded surface display image of a virtual colonoscopy study

Three-dimensional SSD has also been proposed for assessing the biliary tree. CT cholangiography is possible using an appropriate contrast agent specific for the biliary tract. With proper segmentation, SSD-VR can then be used to perform virtual cholangiography.[14,15]

The principle limitation is the presence of the “stair-step” artifact attributed to discontinuities in the scanner dataset. Since these discontinuities are due to nonvolumetric data acquisition, this artifact is not frequently encountered with MDCT.

### Curved plane reconstructions

**Figure 8:**
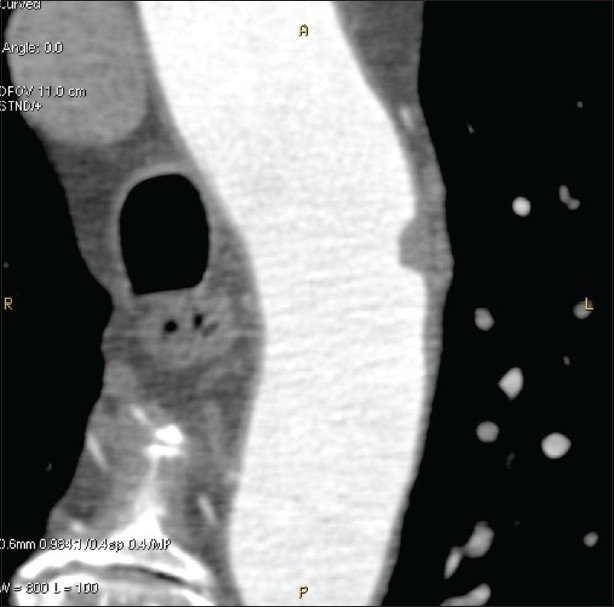
Curved plane reconstruction of the aortic arch shows a small filling defect (arrow) in its lumen

**Figure 9 (A-D):**
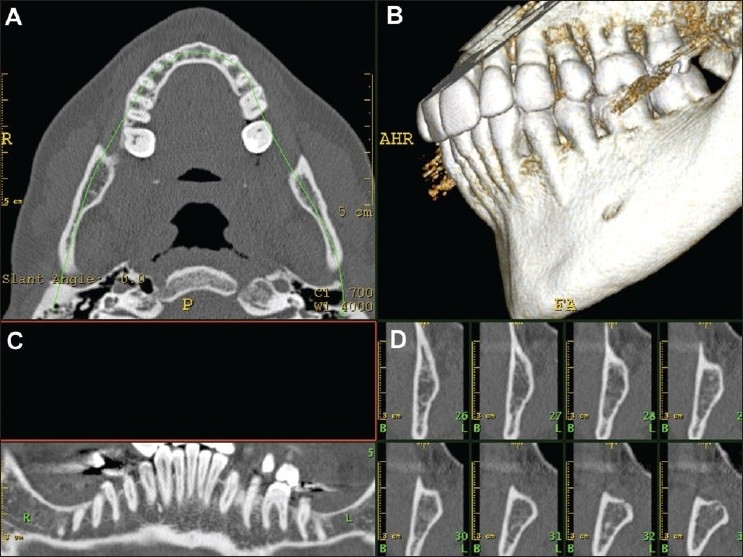
Dental CT reconstructions. By drawing a manual trace on an axial CT scan (A), it is possible to obtain shaded surfaced display volume rendered (B), curved multiplanar reconstruction (C), and paraxial (D) images

Curved plane reconstructions (also called, curved multiplanar reconstructions or curved MPR) are a subcategory of multiplanar reconstructions; instead of representing a plane oriented in one specific direction, they display all voxels contained in a user-selectable curved surface as a single bidimensional image. This allows the user to follow winding structures in their entirety along their natural path of development in a single image. This technique is particularly apt for the study of the vascular system. It is used to display a winding vessel as a straight line and thereby facilitates the identification of vascular defects, stenoses, and dilatations[16] [Figure 8]. It is also well suited to depict the winding course of the mandibular canal, along with the nearby structures, in dental applications. This method is often used to obtain zoomed images that are cut perpendicularly to the canal course, depicting dental roots, and their relationship with the mandibular nerve [Figure 9].

**Figure 10 (A,B):**
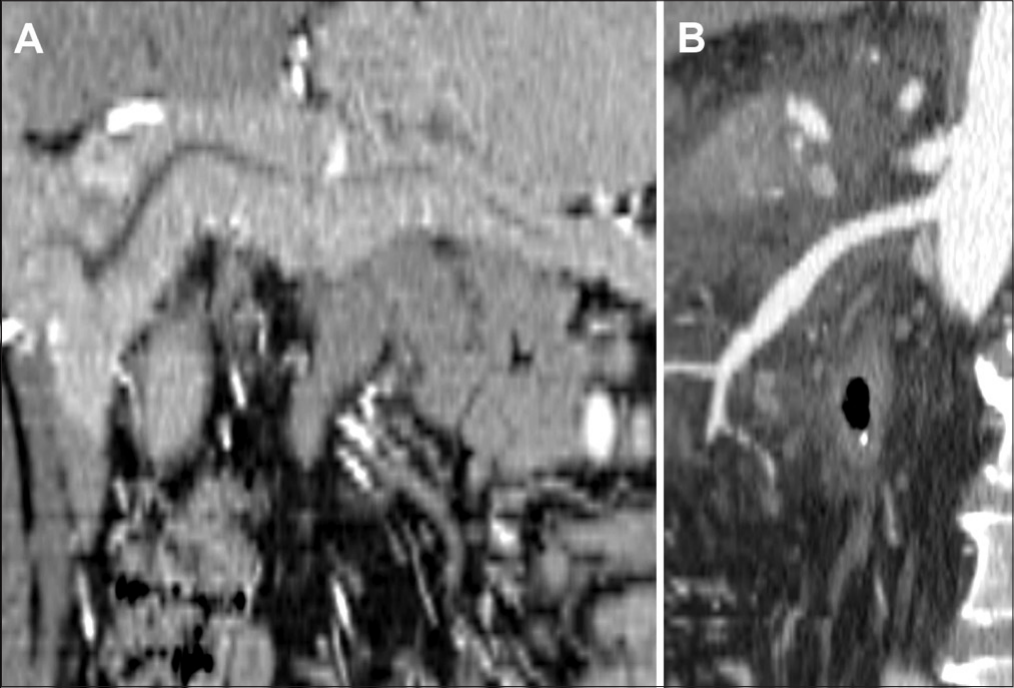
Curved multiplanar reformatted images through the pancreas show the main pancreatic duct (arrow in A) and the superior mesenteric artery (arrow in B)

One of the most noteworthy applications of this technique is in the preoperative CT staging of pancreatic carcinoma. In such staging, it is crucial to determine the involvement of the adjacent vasculature to determine surgical resectability and curved plane reconstructions have proven to be an excellent tool for this [Figure 10].[17]

## Conclusion

In this introductive overview of CT reconstructions, we have considered the most commonly used techniques and their usefulness in some common routine-practice situations. Other volumetric reconstruction techniques exist, but their use is currently less common in clinical practice and disputed in literature. However, the field of CT reconstruction techniques is in continuous evolution[18] and newer, even more sophisticated techniques, currently restricted to the research environment, are likely to emerge as technology and diagnostic workstations evolve. Nonetheless, a small number of everyday rendering techniques can make a big difference in rapidly detecting and properly diagnosing relatively common conditions such as subtle lumen thrombosis, airways stenosis, post-surgery changes, exophytic cancer of hollow organs, and traumatic disorders. A basic knowledge of these rendering techniques and an appreciation of how they can fit into clinical practice, as well as an idea of how the final images are reconstructed from the original data, are nowadays mandatory skills for every professional working with CT scan images.
